# Perceived Didactic Curricular Effectiveness of In-Person vs. Virtual Formats amongst Fourth-Year Dental Students

**DOI:** 10.3390/dj10040060

**Published:** 2022-04-02

**Authors:** Robert D. Bowers, Lance Brendan Young, Carissa L. Comnick, Hariyali P. Kasundra, Christopher A. Barwacz

**Affiliations:** 1 Department of Family Dentistry, College of Dentistry, University of Iowa, Iowa City, IA 52242, USA; chris-barwacz@uiowa.edu; 2 Department of Preventive and Community Dentistry, College of Dentistry, University of Iowa, Iowa City, IA 52242, USA; brendan-young@uiowa.edu; 3 Division of Biostatistics and Computational Biology, College of Dentistry, University of Iowa, Iowa City, IA 52242, USA; carissa-comnick@uiowa.edu; 4 Independent Researcher, Houston, TX 77030, USA; hariyali-kasundra@uiowa.edu

**Keywords:** dental education, COVID-19 pandemic, undergraduate dental education, online learning, blended learning

## Abstract

Purpose: The COVID-19 pandemic altered the methodologies of dental education delivery, resulting in both immediate and more enduring changes. To assess student perceptions of learning effectiveness, graduating dental students from the class of 2020 were surveyed to identify student comfort with technology and content retention, individual motivation and mental focus, and access to resources pertaining to an abrupt transition to a virtual learning didactic seminar approach in March 2020. Methods: a voluntary, 18-question electronic survey was distributed to fourth-year dental students prior to graduation to assess perceptions of learning outcomes and preferences of a virtual seminar format relative to previous in-person didactic seminars experienced. Results: 34 of 80 dental students (42.5%) completed the electronic survey. Comfort and retention of concepts through virtual learning were reported ≥ by 91% and 85% of the respondents, respectively. Increased distractions and multitasking were reported with virtual learning in 56% and 71%, respectively. Desires to have all teaching conducted through virtual learning platforms was reported at 21%. Conclusions: the positive student responses obtained when comparing virtual to in-person seminars in the survey assessment demonstrates the long-term potential for such delivery modalities to be intentionally incorporated into an evolving predoctoral curriculum in a hybrid nature.

## 1. Introduction

Predoctoral dental education is typically conducted in heterogenous settings to optimally facilitate educational objectives and curricula, including in-person didactic seminars, pre-clinical laboratory and simulation exercises, and live patient-based clinical experiences. However, such educational settings have a high propensity to transmit aerosols that may contain the novel SARS-CoV-2 coronavirus amongst asymptomatic students, faculty, staff, and patients, whether in a passive modality through respiratory droplet dissemination, or actively as a result of aerosol-generating dental procedures [1,2,3,4,5]. The pandemic resulted in abrupt changes not only to every facet of society at large, but also more specifically regarding how dental education could safely continue to be delivered during the onset of the pandemic [6]. In the early stages of the pandemic, dental schools adopted virtual platforms to alternatively provide didactic and clinical education in order to protect students, patients, as well as faculty and staff members. Most dental schools suspended direct student patient-care clinical activities following guidance by the American Dental Association (ADA) and Center for Disease Control (CDC) advising dentists to solely treat patients with emergency needs [7,8]. As clinical experience is a critical component of dental education, faculty and administrators were faced with a new educational challenge: keeping dental students motivated, engaged, and meeting educational benchmarks in a virtual learning environment [9].

Prior to the COVID-19 era, the use of virtual learning was increasing; however, it was not the predominate educational approach in higher education. The National Center for Education survey statistics from 2019 found that 37% of students enrolled at degree-granting postsecondary institutions had reported taking at least 1 virtual education course [10]. Subsequently to COVID-19, a growing number of available virtual learning platforms including Moodle, Zoom, Google platforms (Meet, Hangout, Classroom), Skype, Webex, etc. are being widely implemented to facilitate virtual learning [11,12]. Such platforms for educational delivery are increasingly being utilized as a means to deliver educational curricula while facilitating social distancing, especially in lieu of COVID-19 variants that have repeatedly thwarted the full return to in-person education. 

Over the past year, vaccine and therapeutic measures implemented against COVID-19 have assisted much of society slowly returning, in a fragmented fashion, to baseline pre-pandemic measures. Pertaining to dental education, vaccines, proper personal protective equipment, and additional prophylactic preventive measures (e.g., implementing pre-treatment oral rinses to reduce viral particle load) have facilitated a return to clinical education and patientcare [13]. As such measures continue to evolve, there may be less of an impetus for maintaining didactic coursework in a virtual learning environment unless student preferences or learning outcomes can demonstrate tangible benefits for its maintenance and further implementation.

Between 23 March and 29 May 2020, fourth year predoctoral dental students at the University of Iowa immediately transitioned from daily in-person didactic seminars to a virtual learning seminar format called Grand Rounds. The virtual Grand Rounds consisted of faculty-facilitated case presentations, open topic discussions, and live lectures from internal collegiate faculty and invited external lecturers which all occurred with the entirety of the graduating dental student class of 2020. This survey study sought to evaluate students’ comfort, motivational levels, relative focus, potential environmental distractions, and overall assessment of content quality regarding the Grand Rounds virtual learning format provided as part of the final 10 weeks of their predoctoral dental education, as compared to a traditional in-person didactic format experienced in the initial 30 weeks of their fourth year in dental school prior to the pandemic’s onset. 

## 2. Materials and Methods

This study was approved by the University of Iowa Institutional Review Board (#202005250, 13 May 2020). A draft survey instrument was developed to query all 80 fourth year predoctoral dental students’ demographics, future practice or educational plans following graduation, prior exposures to and comfort with virtual learning platforms, and perceptions of learning outcomes and preferences using in-person compared to virtual seminar formats. After peer-review and revision from faculty members within the University of Iowa College of Dentistry, a final composition of the survey was approved. The electronic survey tool Qualtrics (Provo, UT, USA) was used to create a digital copy of the final survey and record data metrics from the respondents. The full survey instrument is attached in Appendix A.

Beyond student respondent demographics and historical exposure to virtual learning platforms, the survey was structured to assess three principal thematic domains pertaining to student perceptions of their learning experience in the virtual seminar format, as compared with in-person didactic learning modalities, which were in place prior to mid-March 2020, when COVID-19 social-distancing protocols required adoption of the virtual seminar format:

- Domain #1: The first domain queried student perception of retention of concepts and comfort in the virtual format, as compared with the in-person format;
- Domain #2: The second domain queried student perception of individual motivation and mental focus when in these two seminar formats;
- Domain #3: The third domain assessed student perception of access to didactic resources, including faculty interaction, peer-to-peer interaction, and ability to access didactic content resources in the two formats.

The final portion of the survey instrument assessed preferences for future learning formats based on their didactic seminar format experiences in the 2019–2020 academic year, with an additional open comment field made available to facilitate more granular feedback on their assessment.

A letter detailing the objective of the study and a link to complete the survey via Qualtrics were e-mailed to each fourth year predoctoral student on 18 May 2020. Non-respondents and unfinished respondents were sent two follow-up e-mail reminders via Qualtrics, for a total of three e-mails, until 29 May 2020. If no response was obtained after these three attempts, the student was considered to be a non-respondent. All fourth year predoctoral students were capable of opting out by responding to any of the e-mails indicating that they did not wish to be contacted further or to participate. Additionally, respondents had the option to either selectively answer some or all of the questions from the survey once they began. Any requests not to be contacted again were respected by the research team and no further contact was made to query these students. Data was subsequently exported from Qualtrics to R version 4.0.0 software (The R Foundation for Statistical Computing, Vienna, Austria) for descriptive statistical analysis. 

## 3. Results 

The entirety of the univariate statistics concerning the survey responses is displayed in Appendix B.

Survey participation data was gathered from 34 of the 80 students in the graduating dental class of 2020, for a response rate of 42.5%. Genders reported were twenty female, and fourteen male students. A total of 18 (52.9%) students reported having previous experience with virtual seminar coursework for credit. 

Observations relating to the first domain of the survey found 91.1% (31/34) reported being very or somewhat comfortable with virtual learning platforms overall. Strong or moderate agreement was reported by 79.4% (27/34), in which the virtual Grand Rounds seminar format was conducive to their comprehension and learning of didactic concepts. Retention of concepts presented in Grand Rounds was reported to be significantly or moderately better by 35.2% (12/34) and neither better nor worse by 50.0% (17/34). Overall didactic learning style was reported to be more aligned with the virtual Grand Rounds format rather than in-person seminars by 44.1% (15/34), opposed by 35.3% (12/34), and indifference between approaches by 20.6% (7/34) respondents. The likelihood of utilizing recorded seminar content was reported by 70.6% (24/34) to a moderate or higher extent. Preference for all didactic seminars to be completed virtually was reported by 20.6% (7/34), with 11.8% (4/34) stating preferences for all in-person seminars, and 67.6% (23/34) preferring a hybrid approach.

Results from the second domain found that motivation to pay attention during the Grand Rounds seminars to be unchanged by 35.3% (12/34), improved in 29.4% (10/34), and regressed in 35.3% (12/34). The chat box utilized during virtual Grand Rounds seminars enhanced the learning of 82.4% (28/34) and was a source of distraction for 5.9% (2/34) of the respondents. Distraction by external stimuli was reported higher by 55.9% (19/34), with 70.6% (24/34) reporting to have multitasked during Grant Rounds virtual seminars. 

Results from the third domain found 79.4% (27/34) of the respondents were more comfortable or willing to ask questions to the lecturer in the virtual Grand Rounds format. Findings of 29.4% (10/34) of the respondents were more likely to discuss topics with fellow student colleagues through the virtual format, with 47.1% (16/34) reporting no changes from in-person seminars. All (100%) respondents reported having adequate ability to access the necessary computer equipment and internet service to participate in the virtual Grand Rounds activities.

## 4. Discussion

The COVID-19 global pandemic, two years after its inception, continues to place pressures on institutions of higher learning. Dental schools are uniquely vulnerable to curricular disruption, as learning occurs in a multifaceted manner. Beyond a purely didactic curriculum, a large portion of learning occurs via direct patient care, often involving aerosol-generating procedures. Via evidence-based best practices in personal protective equipment and other aerosol mitigation factors, most dental schools have found ways to either fully reopen or are close to capacity. As part of this effort to resume full instruction, various approaches to deliver dental education are being evaluated. The graduating dental class of 2020 represented a unique cohort, in that most of their didactic education was delivered with traditional in-person instruction. Serving as an internal control, their sudden shift to synchronous virtual learning, and their subsequent perceptions of learning in such an environment, serve as informative responses as to whether virtual learning platforms should continue to be implemented when developing didactic curriculums in the post-COVID-19 era. 

Survey responses regarding the first domain criteria demonstrated a clear majority of student responses being comfortable with the Grand Rounds virtual learning platform, pointing toward this being less of a concern for ongoing implementation in higher education. A total of 85% of the respondents reported either improvement or indifference to the virtual learning format on concept retention. Another survey of 145 dental student responses by Hung et al. found that 87.6% reported a high degree of comfort adapting to the virtual learning format with no students reporting being uncomfortable with the virtual learning technology implemented following the initial COVID-19 shutdown [14]. The Online Learning Consortium report in 2016 found 90% of high education students affirmed that their experience on online learning platforms was the same or better than traditional classroom learning experiences. Although a slightly less defined metric, it appears most students believe they are learning better in a virtual learning environment [15]. Although a topic not able to be assessed from the nature of the current study, objective test score outcomes have also supported shifts towards virtual learning modalities. A systematic review and meta-analysis conducted by Pei et al. demonstrated that 13 of the 14 studies meeting inclusion criteria had higher test score outcomes, although not all being statistically significant [16].

Results from the second domain queries provided insights to potential negative impacts to student performance regarding virtual learning. Creating a culture of engagement and focus between a student and faculty is critical in any education design, although it appears to be even further challenged in the virtual learning space. Roughly a third of the survey respondents noted a regression in attention span, further corroborated by over two-thirds having reported multitasking during the Grant Rounds virtual seminars. Conflicting reports between faculty and student perceptions have been noted in other health professional feedback surveys following shifts to virtual learning platforms. Findings by Vandenberg et al. noted differing perspectives to psychosocial barriers through Zoom by nursing faculty and students. Statistically significant differences (U = 414.00, *p* = 0.005) in which students reported higher degrees of psychosocial barriers on a Likert scale related to Zoom virtual learning in comparison to their faculty’s perceptions [17]. This highlights that faculty perception of the virtual learning environment should not be assumed the same as what the students report to have experienced, a problem that could be anticipated in any learning environment yet likely further exacerbated virtually.

The chat box feature was reported to enhance learning in most students as well as being a minimal source of distraction. Other engagement tools also appear to be vital to incorporate into virtual learning to improve attention span and motivation due to the overall potential declines noted through virtual learning. Regular breakout sessions appear to be a commonly implemented solution to these issues. Khalid et al. identified positive modulating factors in breakout room designs affecting student comfort, productivity, and learning outcomes in an undergraduate course survey. Findings that small groups (2–3 students), short durations, specific tasks assigned, and visits from their instructor improved outcomes in the domains noted above [18].

The immediate shift to online education at the start of the COVID-19 pandemic necessitated students having adequate access to required resources to leverage such digital platforms for online learning. According to the American Community Survey (ACS) completed in 2019, 95% of households with children between the ages of 3 and 18 years old had some form of home internet access [10]. Furthermore, recent survey statistics have shown a relationship between education level and family income that was directly proportional to home internet access [19]. Considering the education status of the dental student population surveyed, it was not surprising to see that 100% of the students did not report having any significant issues with computer or online access. 

For electronic, virtual learning environments, education can be delivered in two ways: synchronous or asynchronous remote learning. For synchronous learning modalities, students interact with instructors in real time through group chats, breakout rooms, video conferencing, live webinars, etc. With asynchronous settings, instructors deliver educational materials via pre-recorded seminars and online forums in which students learn from these resources at a time and pace of their choosing. The virtual Grand Rounds format implemented during the last 10 weeks of education for the Class of 2020 surveyed was orchestrated through various methods of synchronous virtual learning delivery. Important differences can be noted between synchronous and asynchronous relating to success and student preferences between the delivery methods. A study conducted by Chen et al. demonstrated that students preferred synchronous interactive virtual learning with live questions and answers, as well as small group discussions, showing improved student engagement and learning outcomes reported [20]. However, barriers and challenges for students also exist with virtual learning, including access to stable internet connection, environmental disturbances, and financial burden [21]. Given these factors at play concerning the delivery of live virtual sessions, asynchronous learning can be similarly effective for self-motivated students that have an ability to complete their work on their own established schedule to better manage the aforementioned challenges. Additional potential advantages to students concerning virtual learning platforms with an asynchronous structure is the ability to repeatedly view lectures either partially, or in their entirety, as well as varying the lecture’s playback speed if deemed beneficial in review. Yeung et al., reported students having strong student preferences to having access to recorded lecture content in higher education. Their survey further demonstrated that over half of students will access recorded lecture content during the period of an in-person course, and that individuals that missed lectures more often were less likely to access the lecture content than those who regularly attended lectures [22]. These findings correlate to a general perception in which the lecture recordings can provide reassurance to students that miss an occasional lecture from illness or scheduling conflicts rather than encouraging lack of attendance. Given the versatility of virtual learning, it is easy to overlook the identified benefits of in-person didactic learning especially regarding clinical based education. Recent literature regarding objective structured clinical examinations (OSCEs) and quizzes in a physical examination course found statistically significant higher testing scores in students that attended in-person lectures regularly (>50%) versus students that more often watched virtually instead [23]. The test score differences were smaller in nature and could be attributed to other factors regarding students who prefer one method to another and not the learning style itself. A factor that has been noted to potentially be even more of a variable regarding learning outcomes beyond the mode of delivery is the class size itself [24]. Synchronous virtual learning interactions can be similarly compared to in-person classroom size related challenges regarding the likelihood of peer-peer or student-instructor interactions to occur. Newer technological tools discussed that can be implemented in synchronous learning delivery (i.e., breakout room designs) can potentially help overcome interaction concerns in courses with larger enrollments.

A student’s overall self-motivation has been highlighted as a key factor to success regarding why certain students succeed while others face more challenges in virtual learning formats. Higher student motivation has been shown to improve when student peer to peer interaction along with regular faculty interaction is integrated with virtual learning structure [25]. Contrasting responses were found in the current survey regarding the willingness of a student to ask questions or engage with faculty in comparison to their motivation to interact with fellow peers during Grand Rounds. Approximately 4 out of 5 students reported feeling more comfortable engaging with faculty during Grand Rounds, while the motivation to interact with their fellow peers concerning the content was mostly unchanged. Systematically incorporating breakout sessions or student messaging boards into a virtual learning curriculum could facilitate improved peer engagement reports. Breakout rooms were not incorporated into the virtual Grand Rounds structure although in future applications of synchronous learning this feature would likely help with the challenges regarding distractions by some students found in the current survey. Identifying a source for increasing motivation of students to interact with fellow peers on virtual learning platforms may also lie in the ability to participate anonymously, a challenge to facilitate in both in-person and virtual learning environments. Jong et al. found group discussion with anonymity facilitated higher rates of participation between undergraduate university students than group discussions face to face. This is based on the idea that discussions between students often rely on the more knowledgeable participants to provide acceptable answers resulting in shorter deliberations and less input from the majority of students [26]. A design characteristic that can be easily incorporated into a virtual learning platform with a new profile of technological capabilities through investments made commercially in virtual learning software spaces throughout the COVID-19 pandemic. 

A small sample size of respondents is a limitation noted relating to the survey’s external applicability. The response rate was relatively high considering average participation rates for course evaluation surveys, yet further selection effect influence may exist concerning the portion of students participating having had differing reflections on their experiences than to those who chose not to participate. Future investigations identifying which specific virtual learning platforms students have previously experienced and their potential impact on virtual learning outcomes is a topic of interest to continue to facilitate evidence-based improvement to future curriculum planning in the post-COVID-19 era. The limited timeframe available to construct the survey instrument due to the early pandemic uncertainties in dental education presented a limiting factor regarding the typical validation process followed regarding a more novel approach assuring the stability and consistency of data being collected. Future studies will have sufficient time to ideally validate survey questions and design prior to dissemination. As students build experience and exposure time to virtual learning platforms, future investigations identifying changes in perceptions and performance following prolonged exposure to virtual learning warrants further attention as well. The Class of 2020 served as an “internal control” due to their abrupt transition from traditional in-person seminars to virtual seminars in the span of two-weeks. Distinct from typical course evaluation surveys, the virtual Grand Rounds seminar format involved diverse seminar content, structure, and instructors, all factors that have potential impact on student feedback which were partially mitigated by the virtual learning format of Grand Rounds.

## 5. Conclusions

As vaccines, treatments, and general safeguarding practices targeting COVID-19 transmission have become more widely available and understood, higher education institutions have begun to make transitions back to in-person classes on campuses. Findings regarding the three domains in the current survey designed to identify student assessments and preferences highlighted the many potential ongoing benefits virtual learning brings to dental education regarding improved concept retention without notable concerns for access to necessary resources to facilitate this delivery method. Further knowledge was gained concerning the challenges virtual learning presents for some dental students regarding sustained mental focus and lessened peer to peer interaction than in in-person formats. A future shift toward a hybrid didactic learning format should be considered in dental education based on generally improved assessments seen in the current and recent higher education surveys including important variables regarding a given student’s comfort operating on virtual learning platform and having reliable access to necessary equipment resources. Continuing to seek out factors relating to the success of virtual learning in future studies, while correlating existing literature data regarding differences found pre- and post-pandemic related forced education shifts, will provide improved insight to these issues and help advance ongoing hybrid education delivery in dental education. 

## Data Availability

The data presented in this study are available on request from the corresponding author.

## Appendix A

Family Dentistry COVID-19 Virtual Grand Rounds Educational Perception Survey Questions

1. What is your gender?
  - Male
  - Female
2. Please select one option below which most closely describes your immediate professional plans in your first year following graduation from dental school:
  - Associateship in private practice
  - Ownership/partnership (buy-in) in private practice
  - DSO/Corporate dental practice (i.e., Aspen, Heartland, Pacific, etc.)
  - Specialty residency program
  - AEGD/GPR residency program
  - Public health clinic (FQHC or other)
  - Military dental service
  - Other (open text box)
3. Prior to dental school, did you take a didactic lecture/seminar course entirely virtually/digitally for credit? (note: not including laboratory or exams)
  - Yes
  - No
4. Overall, I would rate my personal comfort-level with digital/virtual learning platforms as:
  - Very comfortable
  - Somewhat comfortable
  - Neither comfortable or uncomfortable
  - Somewhat uncomfortable
  - Very uncomfortable
5. Overall, the virtual COVID-19 Grand Rounds seminar format was conducive to my comprehension and learning of didactic concepts pertaining to my dental education prior to graduation.
  - Strongly agree
  - Moderately agree
  - Neither agree or disagree
  - Moderately disagree
  - Strongly disagree
6. I feel that my retention of concepts presented by the faculty lecturers via the virtual Grand Rounds seminar format, as compared with traditional in-person seminars was:
  - Significantly better
  - Moderately better
  - Neither better or worse
  - Moderately worse
  - Significantly worse
7. I felt more comfortable and/or willing to ask questions of the presenter or other Faculty in the virtual Grand Rounds seminar format, as compared with traditional in-person seminars.
  - Strongly agree
  - Moderately agree
  - Neither agree or disagree
  - Moderately disagree
  - Strongly disagree
8. I felt more motivated to discuss topics covered in the virtual Grand Rounds seminar format with my fellow student colleagues, as compared with traditional in-person seminars.
  - Strongly agree
  - Moderately agree
  - Neither agree or disagree
  - Moderately disagree
  - Strongly disagree
9. I felt more motivated to pay attention to the faculty lecturer in the virtual Grand Rounds seminar format, as compared with traditional in-person seminars.
  - Strongly agree
  - Moderately agree
  - Neither agree or disagree
  - Moderately disagree
  - Strongly disagree
10. I felt the chat box enhanced my learning during the virtual Grand Rounds seminar format, as compared with traditional in-person seminars where questions are voiced verbally.
  - Strongly agree
  - Moderately agree
  - Neither agree or disagree
  - Moderately disagree
  - Strongly disagree
11. I felt more distracted by the chat box during the virtual Grand Rounds seminar format, as compared with traditional in-person seminars, where questions are voiced verbally.
  - Strongly Agree
  - Moderately agree
  - Neither agree or disagree
  - Moderately disagree
  - Strongly disagree
12. I felt more distracted by external/peripheral stimuli (e.g., distracting noises, pets, other people, electronics, etc.) during the virtual Grand Rounds seminar format, as compared with traditional in-person seminars.
  - Strongly agree
  - Moderately agree
  - Neither agree or disagree
  - Moderately disagree
  - Strongly disagree
13. Please choose one option below that best summarizes the nature of your mental focus during the virtual Grand Rounds seminar format:
  - I focused the majority of my attention span to the Grand Rounds content, with minimal distraction or regard to other stimuli/activities
  - I focused some of my attention span to the Grand Rounds content, but also multitasked to other activities or stimuli
  - I focused minimally on the Grand Rounds content, often multitasking or prioritizing other activities or assignments and periodically checking back in on the Grand Rounds seminar
14. My didactic learning style is more aligned with the virtual Grand Rounds seminar format, as compared with in-person seminars:
  - Strongly agree
  - Moderately agree
  - Neither agree or disagree
  - Moderately disagree
  - Strongly disagree
15. If used during the academic year, I would be likely to revisit or re-watch virtual seminars that were given and recorded electronically.
  - Strongly agree
  - Moderately agree
  - Neither agree or disagree
  - Moderately disagree
  - Strongly disagree
16. I felt that I had proper access to necessary computer hardware, software, and internet service to facilitate my learning during the virtual Grand Rounds seminar format.
  - Strongly agree
  - Moderately agree
  - Neither agree or disagree
  - Moderately disagree
  - Strongly disagree
17. Since you have had both in-person, and real-time virtual seminar formats, please select the option that you would most prefer, assuming that you were to repeat your D4 didactic curriculum (don’t worry, you’re graduating!) and that COVID-19 was no longer of concern:
  - I would prefer all didactic seminars be real-time virtual
  - I would prefer some didactic seminars to be real-time virtual, and some to be in-person, depending on the topic and/or speaker
  - I would prefer all seminars be in-person
18. General comments (and/or) suggestions related to your FAMD virtual seminar experience:
  __________________________________________________________________________________________________________________

## Appendix B

Univariate Statistics of the Grand Rounds Survey Responses

What is your gender?	
Female	20 (58.8%)
Male	14 (41.2%)
Please select one option below which most closely describes your immediate professional plans in your first year following graduation from dental school:	
Associateship in private practice	13 (38.2%)
Ownership/partnership (buy-in) in private practice	2 (5.88%)
DSO/Corporate dental practice (i.e., Aspen, Heartland, Pacific, etc.)	3 (8.82%)
Specialty residency program	5 (14.7%)
AEGD/GPR residency program	6 (17.6%)
Public health clinic (Federally Qualified Health Center or Other)	2 (5.88%)
Military dental service	2 (5.88%)
Other	1 (2.94%)
Prior to dental school, did you take a didactic lecture/seminar course entirely virtually/digitally for credit? (Note: not including laboratory or exams):	
No	16 (47.1%)
Yes	18 (52.9%)
Overall, I would rate my personal comfort-level with digital/virtual learning platforms as:	
Very comfortable	16 (47.1%)
Somewhat comfortable	15 (44.1%)
Somewhat uncomfortable	3 (8.82%)
Overall, the virtual COVID-19 Grand Rounds seminar format was conducive to my comprehension and learning of didactic concepts pertaining to my dental education prior to graduation.	
Strongly agree	12 (35.3%)
Moderately agree	15 (44.1%)
Neither agree nor disagree	4 (11.8%)
Moderately disagree	1 (2.94%)
Strongly disagree	2 (5.88%)
I feel that my retention of concepts presented by the Faculty lecturers via the virtual Grand Rounds seminar format, as compared with traditional in-person seminars was:	
Significantly better	6 (17.6%)
Moderately better	6 (17.6%)
Neither better or worse	17 (50.0%)
Moderately worse	5 (14.7%)
I felt more comfortable and/or willing to ask questions of the Guest Lecturer or Faculty in the virtual Grand Rounds seminar format, as compared with traditional in-person seminars.	
Strongly agree	13 (38.2%)
Moderately agree	14 (41.2%)
Neither agree nor disagree	4 (11.8%)
Moderately disagree	3 (8.82%)
I felt more motivated to discuss topics covered in the virtual Grand Rounds seminar format with my fellow student colleagues, as compared with traditional in-person seminars:	
Strongly agree	6 (17.6%)
Moderately agree	4 (11.8%)
Neither agree nor disagree	16 (47.1%)
Moderately disagree	7 (20.6%)
Strongly disagree	1 (2.94%)
I felt more motivated to pay attention to the Faculty lecturer in the virtual Grand Rounds seminar format, as compared with traditional in-person seminars.	
Strongly agree	2 (5.88%)
Moderately agree	8 (23.5%)
Neither agree nor disagree	12 (35.3%)
Moderately disagree	7 (20.6%)
Strongly disagree	5 (14.7%)
I felt the chat box enhanced my learning during the virtual Grand Rounds seminar format, as compared with traditional in-person seminars where questions are voiced verbally.	
Strongly agree	17 (50.0%)
Moderately agree	11 (32.4%)
Neither agree nor disagree	5 (14.7%)
Moderately disagree	1 (2.94%)
I felt more distracted by the chat box during the virtual Grand Rounds seminar format, as compared with traditional in-person seminars, where questions are voiced verbally.	
Moderately agree	2 (5.88%)
Neither agree nor disagree	6 (17.6%)
Moderately disagree	13 (38.2%)
Strongly disagree	13 (38.2%)
I felt more distracted by external/peripheral stimuli (e.g., distracting noises, pets, other people, electronics, etc.) during the virtual Grand Rounds seminar format, as compared with traditional in-person seminars.	
Strongly agree	7 (20.6%)
Moderately agree	12 (35.3%)
Neither agree nor disagree	6 (17.6%)
Moderately disagree	7 (20.6%)
Strongly disagree	2 (5.88%)
Please choose one option below that best summarizes the nature of your mental focus during the virtual Grand Rounds seminar format:	
I focused the majority of my attention span to the Grand Rounds content, with minimal distraction or regard to other stimuli/activities	10 (29.4%)
I focused some of my attention span to the Grand Rounds content, but also multitasked to other activities or stimuli	21 (61.8%)
I focused minimally on the Grand Rounds content, often multitasking or prioritizing other activities or assignments and periodically checking back in on the Grand Rounds seminar	3 (8.82%)
My didactic learning style is more aligned with the virtual Grand Rounds seminar format, as compared with in-person seminars:	
Strongly agree	9 (26.5%)
Moderately agree	6 (17.6%)
Neither agree nor disagree	7 (20.6%)
Moderately disagree	10 (29.4%)
Strongly disagree	2 (5.88%)
If used during the academic year, I would be likely to revisit or re-watch virtual seminars that were given and recorded electronically.	
A great deal	14 (41.2%)
A moderate amount	10 (29.4%)
A little	6 (17.6%)
None at all	4 (11.8%)
I felt that I had proper access to necessary computer hardware, software, and internet service to facilitate my learning during the virtual Grand Rounds seminar format.	
Strongly agree	26 (76.5%)
Moderately agree	8 (23.5%)
Since you have had both in-person, and real-time virtual seminar formats, please select the option that you would most prefer, assuming that you were to repeat your D4 didactic curriculum (don’t worry, you’re graduating!) and that COVID-19 was no longer of concern:	
I would prefer all didactic seminars be real-time virtual	7 (20.6%)
I would prefer some didactic seminars to be real-time virtual, and some to be in-person, depending on the topic and/or speaker	23 (67.6%)
I would prefer all seminars be in-person	4 (11.8%)
